# ‘Death and Axes’: Unexpected Ca^2+^ Entry Phenologs Predict New Anti-schistosomal Agents

**DOI:** 10.1371/journal.ppat.1003942

**Published:** 2014-02-20

**Authors:** John D. Chan, Prince N. Agbedanu, Mostafa Zamanian, Sarah M. Gruba, Christy L. Haynes, Timothy A. Day, Jonathan S. Marchant

**Affiliations:** 1 Department of Pharmacology, University of Minnesota, Minneapolis, Minnesota, United States of America; 2 Department of Biomedical Sciences, Iowa State University, Ames, Iowa, United States of America; 3 Department of Chemistry, University of Minnesota, Minneapolis, Minnesota, United States of America; 4 The Stem Cell Institute, University of Minnesota, Minneapolis, Minnesota, United States of America; University of Pennsylvania, United States of America

## Abstract

Schistosomiasis is a parasitic flatworm disease that infects 200 million people worldwide. The drug praziquantel (PZQ) is the mainstay therapy but the target of this drug remains ambiguous. While PZQ paralyses and kills parasitic schistosomes, in free-living planarians PZQ caused an unusual axis duplication during regeneration to yield two-headed animals. Here, we show that PZQ activation of a neuronal Ca^2+^ channel modulates opposing dopaminergic and serotonergic pathways to regulate ‘head’ structure formation. Surprisingly, compounds with efficacy for either bioaminergic network in planarians also displayed antischistosomal activity, and reciprocally, agents first identified as antischistocidal compounds caused bipolar regeneration in the planarian bioassay. These divergent outcomes (death versus axis duplication) result from the same Ca^2+^ entry mechanism, and comprise unexpected Ca^2+^ phenologs with meaningful predictive value. Surprisingly, basic research into axis patterning mechanisms provides an unexpected route for discovering novel antischistosomal agents.

## Author Summary

Schistosomiasis (Bilharzia) is one of the most burdensome parasitic worm infections, encumbering third world economies with an annual loss of several million disability-adjusted life years. The key treatment for schistosome infections is the drug praziquantel but the mechanism of action of this drug remains controversial hampering targeted development of next generation antischistosomal agents. Here we provide fresh insight into the signaling pathways engaged by PZQ, by resolving commonalities in the action of PZQ with the process of regenerative signaling in free-living planarian flatworms. A similar calcium-dependent network is engaged in both model systems, but with divergent phenotypic outcomes. This relationship provides predictive insight such that basic research on signaling pathways involved in tissue regeneration reveals novel drug leads for schistosomiasis, and reciprocally schistosomal drug screens reveal targets involved in regenerative signaling. We believe this phenology will be helpful for uncovering new antischistosomal drug targets by exploiting broader vulnerabilities within the PZQ interactome.

## Introduction

Over a third of the world's population is estimated to be infected with parasitic worms. One of the most burdensome infections underpins the neglected tropical disease schistosomiasis (Bilharzia), caused by parasitic flatworms of the genus *Schistosoma*. The debilitating impact of schistosomiasis results from the host's immune response to schistosome eggs, which are deposited in prolific numbers in the liver, intestine and/or bladder where they elicit granuloma formation and fibrosis [1]. Clinical outcomes span gastrointestinal and liver pathologies, anaemia, undernutrition, growth retardation, genitourinary disease and a heightened risk for co-morbidities. This burden encumbers third world economies with an annual loss of several million disability-adjusted life years [2]–[4].

The key treatment for schistosome infections is the drug praziquantel (PZQ). PZQ is a synthetic tetracyclic tetrahydroisoquinoline derivative discovered over 30 years ago to confer anthelminthic activity [5]–[7] by evoking a spastic paralysis of the adult worms [8]. The low cost (∼$0.07/tablet) yet high cure rate associated with PZQ underpins current strategies for increasing PZQ distribution to reduce the burden of schistosomiasis [9], but obviously continued efficacy of PZQ is critical for the success of these initiatives. From a drug development perspective, it remains problematic that despite three decades of clinical use, the target of PZQ remains ambiguous and synthesized structural derivatives prove consistently less efficacious [5]–[7], [10], [11]. Resolution of the target and effector mechanisms of PZQ would be massively helpful for identifying new drug targets that exploit vulnerabilities within the broader PZQ interactome.

Recently, we have attempted to bring fresh insight into the mechanism of action of PZQ by studying an unusual impact of this drug on regeneration of a free living planarian flatworm (*Dugesia japonica*), a representative of a model system widely utilized by basic scientists as a model for regenerative biology [12], [13]. This line of investigation grew from the serendipitous finding that PZQ exposure invariably caused regeneration of worms with two heads (‘bipolar’), rather than worms with normal anterior-posterior (‘AP’, head to tail) polarity [14]. The capacity of PZQ to evoke this complete AP axis duplication was phenocopied by several Ca^2+^ signaling modulators, a relationship underpinned by the demonstration of PZQ-evoked Ca^2+^ uptake in native planarian tissue [14], [15]. The tractability of planarians to *in vivo* RNAi methods allowed mechanistic interrogation of various Ca^2+^ entry pathways, and this approach revealed the bipolarizing efficacy of PZQ depended on the expression of neuronal voltage-operated Ca^2+^ channel (Ca_v_1) isoforms [14], [15]. These observations were intriguing in the context of schistosome biology, as PZQ is well documented to cause Ca^2+^ entry in schistosomes [8], [16], [17] and PZQ has been shown to activate Ca^2+^ entry via modulation of a heterologously expressed schistosome Ca_v_ accessory subunit [18], [19]. But how Ca^2+^ entry engages acute and chronic [20]–[22] downstream signaling pathways in either planarians or schistosomes is less clear, with resolution of this broader PZQ interactome key for identifying new druggable targets and vulnerabilities for chemotherapeutic exploitation [17].

Here, we evidence a Ca^2+^-dependent phenology of PZQ action between these two quite different models. We propose the same Ca^2+^ entry and downstream pathways are engaged by PZQ in planarians and schistosomes, and the mechanistic interrelationship underpinning these different outcomes (death in schistosomes, axis duplication in planarians) augers predictive value for discovery of new anti-schistosomal agents. For example, in planarians, we demonstrate the planarian AP axis duplication phenotype results from coupling of Ca_v_1A activity to bioaminergic signaling. Modulators of regenerative polarity which impact dopaminergic and serotonergic pathways in planarians are effective against schistosomes, and reciprocally recently discovered drug leads active against schistosomes (for example, PKC and GSK3 modulators) regulate AP specification in planarians. As unexpected phenologs [23], this discovery underscores the utility of basic research on axis patterning mechanisms in the tractable planarian system for the discovery of novel antischistosomal drug leads, and more broadly mechanistic insight into the signaling pathways engaged by PZQ, a key human therapeutic.

## Results & Discussion

### Profiling planarian neurotransmitter families

**Figure 1:**
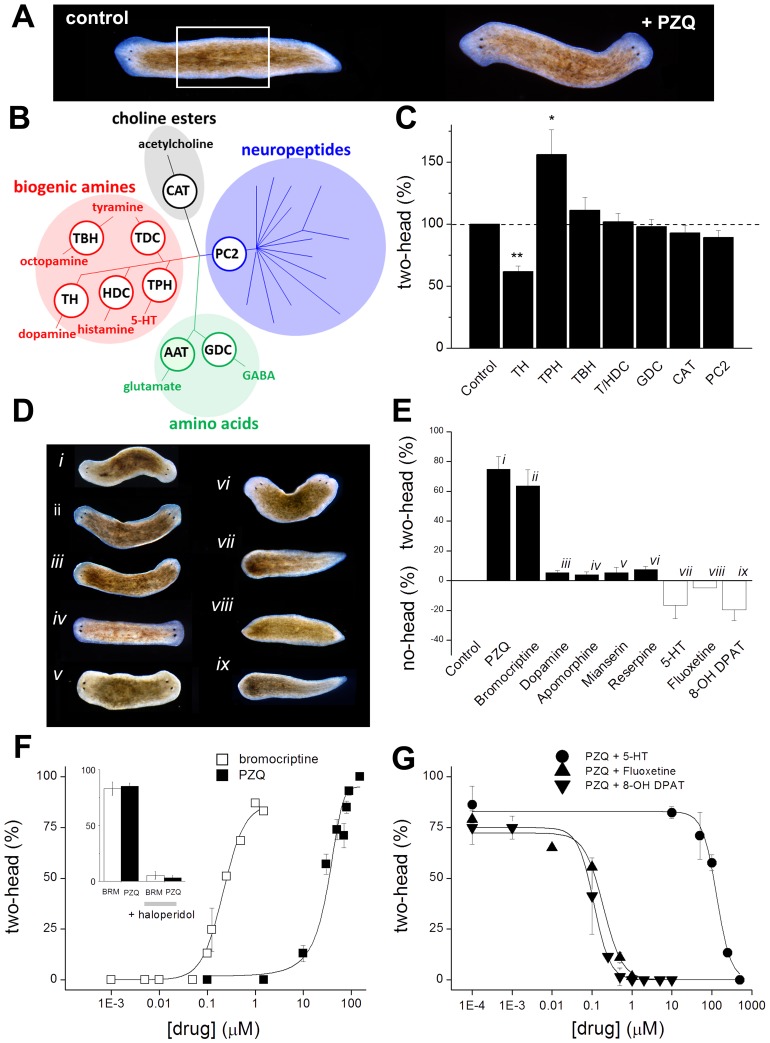
Biogenic amines differentially modulate PZQ evoked bipolarity. (**A**) Anterior posterior (AP) polarity in normal (left, control) and PZQ-treated (75 µM, 48 hrs) *D. japonica* (right) after 7 days of regeneration. This result derived from incubation of trunk fragments (white box, amputation of head and tail structures) in drug-containing solution during early regeneration [16]. (**B**) Diversity of flatworm neurotransmitters. Shading identifies different flatworm neurotransmitter families with branching reflecting molecular diversity. Key synthetic enzymes targeted by RNAi (white circles) were: CAT, choline acetyl transferase; PC2, prohormone convertase 2; GDC, glutamate decarboxylase; TH, tyrosine hydroxylase; HDC, histidine decarboxylase; TDC, tyrosine decarboxylase; TBH, tyramine-β-hydroxylase; TPH, tryptophan hydroxylase. (**C**) Effect of RNAi targeting neurotransmitter synthetic pathways on PZQ-evoked bipolarity, n≥3 independent trials. Abbreviations are as described in ‘B’. (**D**) Pharmacological screening of monoaminergic drugs revealed compounds that promote and inhibit head regeneration. Representative images of regenerative phenotypes observed using (i) PZQ (75 µM), (ii) bromocriptine (1 µM), (iii) dopamine (500 µM), (iv) apomorphine (750 nM), (v) mianserin (10 µM), (vi) reserpine (10 µM), (vii) fluoxetine (2 µM), (viii) 5-HT (1 mM), (ix) 8-OH DPAT (10 µM). In all cases, trunk fragments were treated for 48 hrs. (**E**) Penetrance of monoaminergics at evoking two-headed (black) or no-headed worms (open). (**F**) Bipolarity evoked by PZQ (solid) and bromocriptine (open) was antagonized by haloperidol (inset, co-incubation with 1.5 µM for 24 hrs). (**G**) Inhibition of PZQ-evoked bipolarity (90 µM, 24 hrs) by various concentrations of serotonergic ligands.

Exposure of excised trunk fragments to PZQ caused regeneration of viable, two-headed flatworms (Figure 1A), an effect previously shown to relate to modulation of neuronal voltage-operated calcium (Ca_v_) channels [14], [15]). Given the role of Ca^2+^ entry in synaptic and dendritic exocytosis [24], [25], we hypothesized that PZQ-evoked Ca^2+^ entry impacted neurotransmission and thereby stem cell behavior, consistent with a ‘neurohumoral’ model for regulation of planarian stem cell proliferation proposed two decades ago [26]. To test this idea, we used loss-of-function (*in vivo* RNAi) and pharmacological methods to interrogate whether different planarian neurotransmitters mimicked the PZQ-evoked bipolarity effect. Figure 1B schematically summarizes the major neurotransmitter classes in flatworms [27]–[29], of which neuropeptides predominate by number. A recent characterization of planarian bioactive peptides revealed >50 prohormone genes, the vast majority being neuronally expressed with over 250 discrete peptides generated from these precursors [30]. Further, bioinformatic prediction supports at least 130 planarian neuropeptide targeted G protein coupled receptors [31]. This expansive neuropeptidergic arsenal co-exists with several ‘classic’ neurotransmitter families more familiar to mammalian neurophysiologists. The largest group of these transmitters are the biogenic amines, a group of protonated amines including serotonin, histamine, catecholamines (notably dopamine) as well as tyramine and octopamine, two phenolamines widely used as invertebrate neurotransmitters [27], [32]. Roles for acetylcholine (ACh) and amino acids (glutamate, GABA) are also evidenced [27], [32]. To test the involvement of these different neurotransmitter families as PZQ effectors, we used *in vivo* RNAi to knockdown key enzymes involved in their synthesis. Knockdown of prohormone convertase 2 (PC2, [33]), an enzyme required for motility [34] and neuropeptide processing [30], failed to impact the penetrance of PZQ-evoked bipolarity (Figure 1C). Similarly, knockdown of glutamate decarboxylase (GDC, to decrease planarian GABA levels [35]), and choline acetyltransferase (CAT, to deplete ACh [36]), failed to modulate the penetrance of PZQ (Figure 1C). Negative results were also obtained following RNAi of tyramine-β-hydroxylase (TBH) and tyrosine/histidine decarboxylase (T/HDC). These data were also consistent with the outcomes of pharmacological experiments where application of the phenolamines tyramine and octopamine failed to perturb AP polarity (Table 1).

**Table 1:** Dopaminergic and serotonergic ligands miscue regeneration.

Class	Drug	Activity	Phenotype	[X], source
Neuropeptides	FMRFamide	agonist	-	1 mM,^1^
Neuropeptides	Spantide	antagonist		100 µM,^2^
Adrenergic	Epinephrine	agonist	-	5 mM,^1^
Adrenergic	Norepinephrine	agonist	-	5 mM,^1^
Adrenergic	L-phenylephrine	agonist	-	100 µM,^1^
Adrenergic	Propranolol	antagonist	-	10 µM,^1^
GABAergic	GABA	agonist	-	10 mM,^1^
GABAergic	Piperazine	agonist	-	1 mM,^1^
GABAergic	Baclofen	agonist	-	500 µM,^1^
GABAergic	Carbamazepine	agonist	-	250 µM,^1^
Glutaminergic	L-glutamic acid	agonist	-	500 µM,^1^
Glutaminergic	NMDA	agonist	-	1 mM,^1^
Glutaminergic	AMPA	agonist	-	1 mM,^1^
Glutaminergic	Topiramate	antagonist	-	10 µM,^1^
Cholinergic	Acetylcholine	agonist	-	1 mM,^1^
Cholinergic	Nicotine	agonist	-	1 mM,^1^
**Cholinergic**	**Levamisole**	**agonist**	**2-head (7%)**	**100 µM,** 1
Cholinergic	Muscarine	agonist	-	100 µM,^1^
Cholinergic	Atropine	antagonist	-	500 µM,^1^
Cholinergic	Tubocurarine	antagonist	-	100 µM,^1^
Cholinergic	α-Bungarotoxin	antagonist	-	10 µM,^3^
Biogenic amines	Octopamine	agonist	-	10 mM,^1^
Biogenic amines	Tyramine	agonist	-	100 µM,^1^
Biogenic amines	Histamine	agonist	-	5 mM,^1^
**Dopaminergic**	**Bromocriptine**	**agonist**	**2-head (64%)**	**2 µM,** 1
**Dopaminergic**	**Dopamine**	**agonist**	**2-head (5%)**	**500 µM,** 1
**Dopaminergic**	**Apomorphine**	**agonist**	**2-head (4%)**	**750 nM,** 1
**Dopaminergic**	**Haloperidol**	**antagonist**	**2-head (5%), no-head (4%)**	**5 µM,** 1
**Dopaminergic**	**Bupropion**	**DAT antagonist**	**2-head (10%)**	**1 µM,** 1
**Dopaminergic**	**SKF 38393**	**agonist**	**2-head (4%)**	**50 µM,** 1
**Dopaminergic**	**Trifluoperazine**	**antagonist**	**2-head (15%)**	**10 µM,** 1
Dopaminergic	Pergolide	agonist	-	100 µM,^1^
Dopaminergic	Ropinirole	agonist	-	100 µM,^1^
Dopaminergic	SCH 23390	antagonist	-	10 µM,^1^
Dopaminergic	±Sulpiride	antagonist	-	250 µM,^1^
Dopaminergic	GBR 12909	DAT antagonist	-	1 µM,^1^
**Serotonergic**	**5HT**	**agonist**	**No-head (17%)**	**1 mM,** 1
**Serotonergic**	**8-OH DPAT**	**agonist**	**No-head (20%)**	**10 µM,** 1
**Serotonergic**	**Fluoxetine**	**SSRI**	**No-head (5%)**	**2 µM,** 1
**Serotonergic**	**Mianserin**	**antagonist**	**2-head (6%)**	**10 µM,** 1
Serotonergic	m-CPP	agonist	-	10 µM,^1^
**Multiple targets**	**Reserpine**		**2-head (7.5%)**	**10 µM,** 1
**Multiple targets**	**Amitriptyline**		**2-head (3%)**	**7.5 µM,** 1
**Multiple targets**	**Pindolol**		**2-head (3%)**	**200 µM,** 2
**Multiple targets**	**Bafilomycin A1**		**2-head (3%)**	**10 nM,** 1
**Multiple targets**	**Lobeline**		**2-head (3%), no-head (3%)**	**7.5 µM,** 1
**Multiple targets**	**Clozapine**		**2-head (5%)**	**1 µM,** 1

Results from pharmacological screen investigating the impact of different agents that modify neurotransmission on planarian regenerative polarity. Each ligand within the different classes was tested up to the indicated concentration after first performing toxicity assays to identify the concentration range over which worm viability was unaffected. In each test, drug exposure was for one day using cohorts of n≥30 worms for n = 3 trials. A lack of effect in regenerative polarity is indicated by ‘-’, whereas polarity defects (phenotype and penetrance) are described. Drugs were sourced as follows:

1 Sigma Aldrich,

2 Tocris Bioscience,

3 Invitrogen.

In contrast, results with other biogenic amines were more intriguing – knockdown of tyrosine hydroxylase (TH) attenuated the ability of PZQ to evoke two-headed worms, whereas knockdown of tryptophan hydroxylase (TPH) increased PZQ-evoked bipolarity (Figure 1C). TH is the rate-limiting enzyme of catecholamine synthesis, catalyzing the conversion of tyrosine to L-dihydroxyphenylalanine (L-DOPA), whereas TPH converts tryptophan to 5-hydroxytryptophan, the first step in 5-HT synthesis. Knockdown of TH in *D. japonica* decreases dopamine without impacting 5-HT production [37], while knockdown of TPH decreases 5-HT but not dopamine [38]. These RNAi results suggest that PZQ activity is mimicked by dopaminergic activity (TH RNAi) to promote head regeneration, and this action is opposed by serotonergic signaling (TPH RNAi).

On the basis of this hypothesis, we proceed to screen modulators of dopamine and 5-HT receptors: dopaminergic stimuli should phenocopy the bipolarizing activity of PZQ, while PZQ action should be opposed by serotonergic agonists. While this is a reasonable approach, care must be taken in assuming the specificity of agents established in mammal models transfers to flatworm systems. Flatworms may express more bioaminergic receptors than humans [31], and the few flatworms receptors that have been successfully expressed and pharmacologically profiled [39] underscore the risks of assuming similar drug activities to those assigned in mammals. Keeping this caveat in mind, we nevertheless used a pharmacological approach but accrued evidence with multiple ligands and used secondary validation assays to best mitigate this problem. Below, we first describe results of drug assays assuming specificities based upon mammal data, and then we return to the issue of validating ligand specificity against particular neurotransmitter pathways.

A range of compounds were screened for effects on AP polarity (Table 1), and these investigations yielded the following observations. First, the exclusion of individual neurotransmitter families on the basis of RNAi results (Figure 1C) received further support from pharmacological screening, as most modulators of adrenergic, GABAergic, glutaminergic, histaminergic and cholinergic pathways failed to impact regenerative polarity (Table 1). Second, bromocriptine, a potent D_2_ agonist in mammalian systems, produced two-headed regenerants at high penetrance (maximal effect ∼85±5% bipolar, Figure 1D & 1E), with an EC_50_ of 220 nM compared with an EC_50_ of ∼40 µM for PZQ (Figure 1F). Other dopaminergic modulators yielded a low, but robust, proportion of two headed worms including apomorphine (a non-selective dopaminergic agonist in mammals) and dopamine itself (Figure 1D & 1E). Third, haloperidol, a traditional antipsychotic and known inhibitor of dopaminergic signaling in planaria [40], blocked the bipolarizing activity of both bromocriptine and PZQ (Figure 1F, inset). Fourth, 5-HT blocked head regeneration, an effect observed with 5-HT, the synthetic ligand 8-OH DPAT (a mammalian 5-HT_1A_ agonist) and a serotonin-specific reuptake inhibitor (SSRI, fluoxetine, Figure 1D & 1E), all of which blocked the bipolarizing effect of PZQ (IC_50_ ∼147 µM, 111 nM and 230 nM, Figure 1G). In contrast, mianserin (a 5-HT antagonist in flatworm [41]–[43] and mammalian systems) yielded a small proportion of two-headed worms (Figure 1D&E).

**Figure 2:**
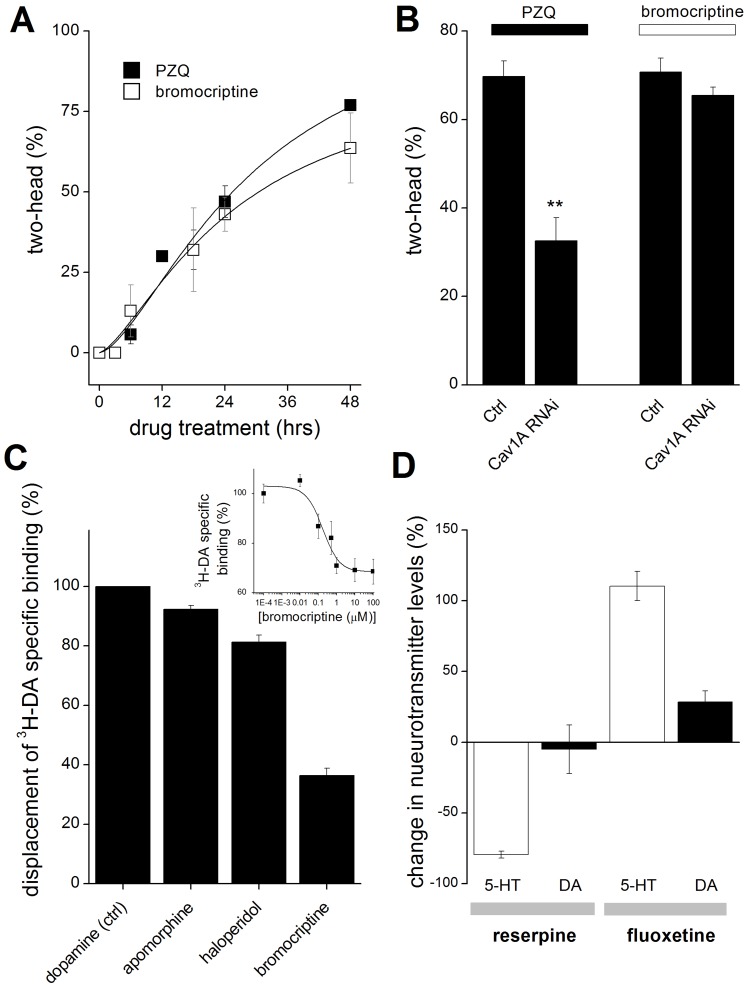
Analysis of drug action and selectivity in the planarian system. **(A)** Similar kinetics of bromocriptine and PZQ-evoked bipolarity. Number of bipolar regenerants after exposure of trunk fragments to PZQ (75 µM, solid) or bromocriptine (1.5 µM, open) for the indicated durations. Fragments were amputated at t = 0. **(B) **Bromocriptine acts downstream of PZQ-evoked Ca^2+^ entry. Ca_v_1A RNAi inhibits PZQ but not bromocriptine-evoked bipolarity. **(C)** Displacement of ^3^H-DA from planarian membrane fractions by various ligands, including dopamine (Ctrl), bromocriptine (10 µM), haloperidol (100 µM), and apomorphine (10 µM). Inset, ^3^H-DA displacement assay at various concentrations of bromocriptine, expressed as a fraction of total specific ^3^H-DA binding. Data represent average of at least three independent replicates. **(D)** Effect of reserpine and fluoxetine on 5-HT and dopamine levels in regenerating trunk fragments. Regenerating trunk fragments were exposed to of either reserpine (10 µM) or fluoxetine (10 µM) for 24 hrs prior to electrochemical HPLC analysis of 5-HT (open) and dopamine (closed) levels. Data represent analyses from multiple samples from at least two independent biological replicates, mean ± standard deviation.

Given the effects of bromocriptine, we further investigated the characteristics of bromocriptine efficacy in planaria. First, bromocriptine exhibited a similar kinetic action to that observed with PZQ (Figure 2A), suggesting a similar action early in regeneration. Second, while knockdown of Ca_v_1A attenuated PZQ-evoked bipolarity, bromocriptine-evoked bipolarity persisted in Ca_v_1A RNAi worms (Figure 2B). This surprising result is consistent with the idea that bromocriptine activation of head signaling pathways occurs downstream of Ca_v_1A function. For example, if PZQ-evoked Ca^2+^ entry [15] activates neurotransmitter release, then the bipolarizing efficacy of bromocriptine should persist at downstream receptors even if Ca^2+^ entry is impaired. Third, given concerns about presumptions of similar pharmacological effects between mammalian and flatworm systems, we investigated whether bromocriptine exhibited affinity for dopaminergic systems in planaria by performing ^3^H-dopamine displacement assays. Specific ^3^H-dopamine binding, defined by complete displacement with cold dopamine (IC_50_ = 1.5±0.5 µM), was inhibited by bromocriptine and other head-promoting agents (haloperidol and apomorphine, Figure 2C). The extent of ^3^H-dopamine displacement by maximally effective concentrations of haloperidol and apomorphine was greater (>80% of specific binding) than observed with bromocriptine (∼40% of specific binding at 10 µM). This indicated bromocriptine may exhibit selectivity for only a subset of dopaminergic targets compared to the broader and more complete binding inhibition observed with the other agents. Finally, we investigated the impact of agents presumed to impact neurotransmitter levels (reserpine, fluoxetine) via HPLC. Figure 2D shows that fluoxetine (a 5-HT reuptake inhibitor on the basis of mammalian and schistosome literature [44], [45]) increased 5-HT levels in regenerating planarian trunk fragments, consistent with the inhibitory effects of 5-HT (and fluoxetine) on head regeneration (Figure 1G). In contrast, reserpine exposure depleted 5-HT in regenerating fragments (Figure 2C), an opposing outcome consistent with the differential polarity effects of these drugs on head regeneration (reserpine vs fluoxetine, Figure 1D&E).

Collectively, these pharmacological data support the model derived from RNAi data (Figure 1C) where dopaminergic signaling mimics and serotonergic activity opposes PZQ action. The distinct phenotypic outcomes of dopaminergic and serotonergic modulation are also consistent with observations that these neurotransmitter networks in planarians are morphologically discrete [28].

### Relevance to schistosomiasis

**Figure 3:**
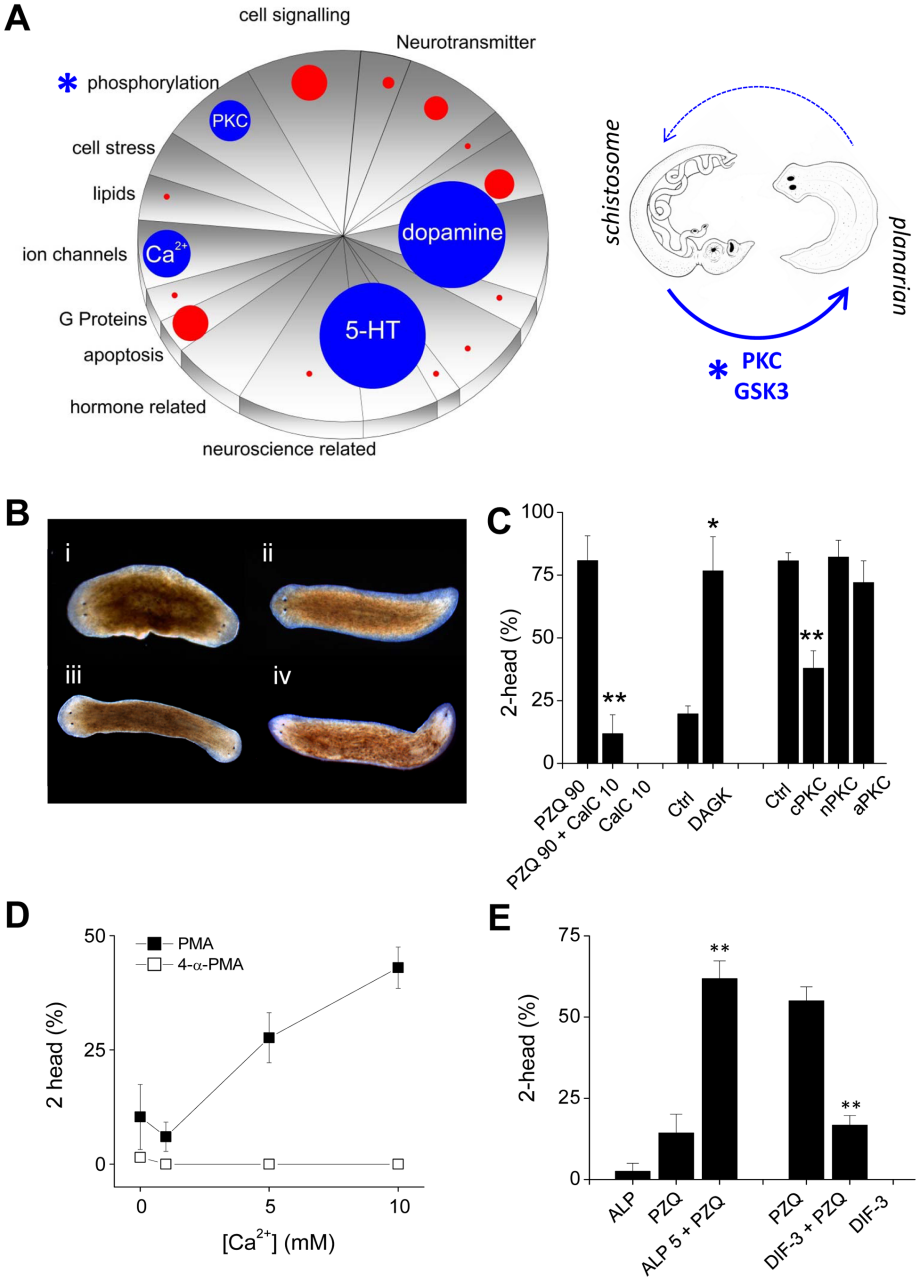
Small molecules efficacious as antischistosomals miscue planarian AP polarity. (**A**) *Left*, Schematic representation of ‘hits’ versus drug representation in *Schistosoma mansoni* drug screens. The number of drug hits in different functional classes (pie chart categories reflect LOPAC_1280_ pharmacological action ontology) were represented as appropriately scaled circles, allowing simple visual inspection of drug categories over/under represented as phenotypic outcomes. Blue circles highlight the top four drug categories (dopaminergics, serotonergics, Ca^2+^ signaling, phosphorylation) and red circles the proportional representation of other classes. For simplicity, drugs with ‘unknown’ mechanism of action classifications, and generalized anti-infective agents were not represented. *Right*, translation of hits (PKC, GSK3) in the phosphorylation category (*) from the schistosome screening literature to the planarian regeneration assay. (**B**) Images of regenerating worms after treatment with PKC modulators: (i) PMA (15 nM), (ii) 4-α PMA (inactive analog, 15 nM), (iii) OAG (100 µM), (iv) PDB (25 µM). (**C**) Involvement of PKC in PZQ-evoked bipolarity. Left, PKC inhibition using calphostin C (10 nM) attenuated PZQ (90 µM) evoked bipolarity. Middle, RNAi of DAGK potentiated low dose PZQ-evoked bipolarity (50 µM). Right, knockdown of a cPKC isoform attenuated PZQ-evoked bipolarity (90 µM). (**D**) Effect of Ca^2+^ on PMA-evoked bipolarity. Effects of indicated Ca^2+^ concentration on the bipolarizing ability of the PKC agonist PMA (solid squares, 15 nM) and the inactive analog 4-α-PMA (open squares, 15 nM). (**E**) The GSK-3 inhibitor ALP (5 µM) potentiated PZQ-evoked bipolarity (25 µM), while the GSK3 activator DIF-3 (1.75 µM) blocked PZQ action (50 µM).

These discoveries piqued our interest since dopaminergic and serotonergic ligands have recently emerged as hits in drug screens against various schistosome life cycle stages [46], [47]. Figure 3A collates examples of recent drug screening data to show how efficacious drug hits are distributed relative to the functional representation of drugs screened [46], [47]. The top three functional categories represent dopaminergic and serotonergic ligands followed by regulators of ion channel activity, notably Ca_v_ channel modulators. This triumvirate parallels the PZQ-engaged components in planarians in this study (bioaminergics, Figure 1) and previously (Ca^2+^ channels, [14], [15]). As such, we propose the distinct phenotypes - PZQ-evoked bipolarity in planarians and PZQ-evoked toxicity against schistosomes - represent unexpected yet orthologous phenotypes (‘phenologs’, [23]) resulting from engagement of the same fundamental Ca^2+^-triggered interactome in each system. Although PZQ-evoked Ca^2+^ entry is evoked via similar mechanisms (Ca_v_1A) it is harnessed in the two organisms to yield differential outcomes (‘death’ versus ‘axes’). The utility of this phenology is its predictive value. As both outcomes derive from the same effector network, basic research on axis patterning in planarians may harbor potential for discovering new agents effective as antischistosomals. This assertion can be tested by asking whether other antischistosomals cause planarian bipolarity, and reciprocally, whether bipolarizing agents in planarians are active against schistosomes.

### Effects of antischistosomals on planarian regeneration

Do other antischistosomal compounds cause planarian bipolarity? To test this, we identified the next most prevalent category from the schistosome drug screening datasets, which was the ‘phosphorylation’ category (Figure 3A). The predominant group of compounds within this category were several drugs that target protein kinase C (PKC), and a couple of singleton kinase inhibitors, including one targeting glycogen synthase kinase-3 (GSK3). We investigated the role of both kinases to resolve any impact on planarian regenerative polarity (Figure 3A). First, the PKC activators phorbol-12-myristate-13-acetate (PMA), phorbol-12,13-dibutyrate (PDB) and oleoyl-acetyl-glycerol (OAG) all produced bipolar worms (penetrance ∼5–55% respectively, Figure 3B), while the PKC inhibitor calphostin C [48] inhibited PZQ-evoked bipolarity (Figure 3C). To complement the pharmacological data with molecular insight, we cloned several planarian PKC isoforms and diacylglycerol kinase (DAGK) and investigated their roles in PZQ-evoked bipolarity by RNAi. Knockdown of DAGK, which opposes PKC activity via the degradation of DAG, potentiated the penetrance of sub-maximal doses of PZQ; while RNAi of a conventional PKC isoform, but not a novel and atypical PKC, attenuated PZQ evoked bipolarity (Figure 3C). The involvement of a Ca^2+^-regulated PKC was also consistent with the observation that the penetrance of PMA in yielding bipolar regenerants was Ca^2+^ dependent (Figure 3D). Similarly, alsterpaullone (ALP), a GSK-3 inhibitor also phenocopied PZQ in regenerative assays, producing a low frequency of two headed worms and synergistically potentiating sub-maximal doses of PZQ (Figure 3E). The small molecule GSK3 agonist DIF-3 [49] displayed the opposing action, inhibiting PZQ-evoked bipolarity (Figure 3E). Therefore, both these targets in the ‘phosphorylation’ category prioritized from the schistosomal screening literature (Figure 3A) were resolved to miscue planarian AP polarity during regeneration.

### Efficacy of bipolarizing compounds against schistosomules

**Figure 4:**
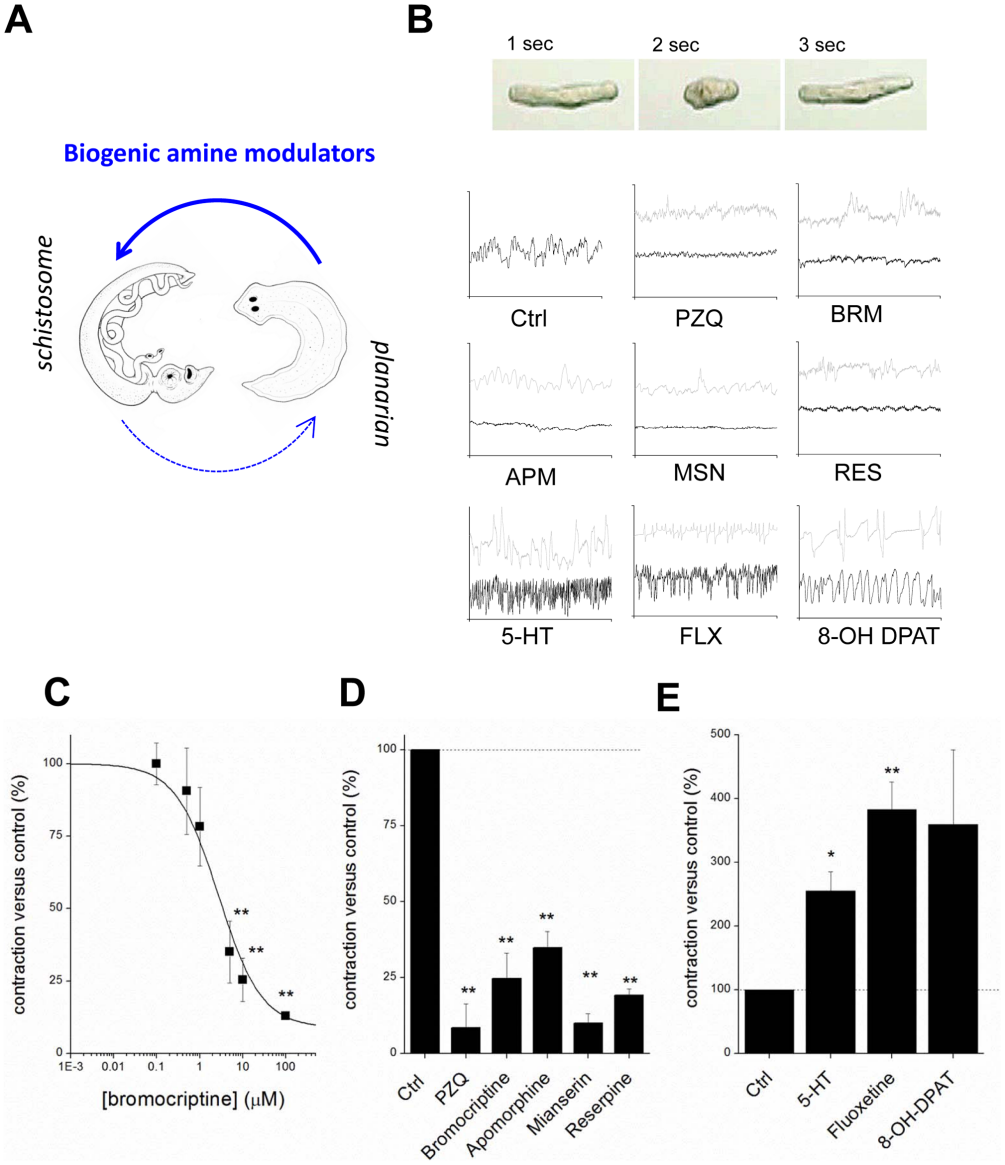
Compounds that miscue planarian polarity regulate schistosomule contractility. (**A**) Compounds transferred from planarian regenerative assay to schistosomule screen. (**B**) *Top*, image sequence showing periodic basal contractile activity of a schistosomule. *Bottom*, body length versus time plots for individual schistosomules treated with small molecules (low dose, grey; high dose, black). Drug concentrations (30 minute exposures) were: bromocriptine (BRM, 1 µM; 10 µM), praziquantel (PZQ, 1 µM; 10 µM), apomorphine (APM, 1 µM; 10 µM), mianserin (MSN, 5 µM; 10 µM), reserpine (RES, 10 µM; 50 µM), 5-HT (10 µM; 100 µM), fluoxetine (FLX, 1 µM; 10 µM), 8-OH DPAT (1 µM; 10 µM). (**C**) Dose-response relationship showing the effects of increasing concentrations of bromocriptine on schistosomule contractility. (**D&E**) Cumulative dataset from experiments such as those shown in ‘B’ for compounds active in the planarian regenerative bioassay parsed into compounds that (D) inhibit and (E) stimulate schistosomule contractility. Drug concentrations were the higher dose of values reported in (B). Dashed line, basal level of contractility.

Are drugs that miscue planarian regeneration deleterious to schistosomes? To investigate this issue, schistosomules (juvenile parasites) were exposed to compounds first identified in planarian regenerative assays (Figure 4A). Schistosomules normally exhibit a basal level of spontaneous contractile activity (Figure 4B), which provides a simple phenotype for assaying drug action and paralysis, an outcome integral to the elimination of schistosome infections [46]. Bromocriptine caused a rapid paralysis of schistosomules, an effect that phenocopied the action of PZQ (Figure 4B). This effect was dose-dependent (Figure 4B&C). Other compounds that yielded planarian bipolarity were also found to impair schistosomule contractility, including apomorphine, mianserin and reserpine (Figure 4B). In contrast, application of exogenous serotonin and other ligands that inhibited planarian head regeneration (e.g. 8-OH DPAT and fluoxetine) resulted in hyperactivity (Figure 4C). Quantification of the action of these agents which inhibited and stimulated schistosomule activity is collated in Figures 4D&E respectively. Therefore, not only were both classes of bioaminergic compounds efficacious against schistosomules, but the dopaminergic and serotonergic ligands evoked divergent phenotypes in each model: paralysis versus hyperactivity (schistosomules), compared with ‘two-headed’ versus ‘no-head’ regenerants (planaria).

### PZQ activity in flatworm models shares a Ca^2+^-dependent phenology

**Figure 5:**
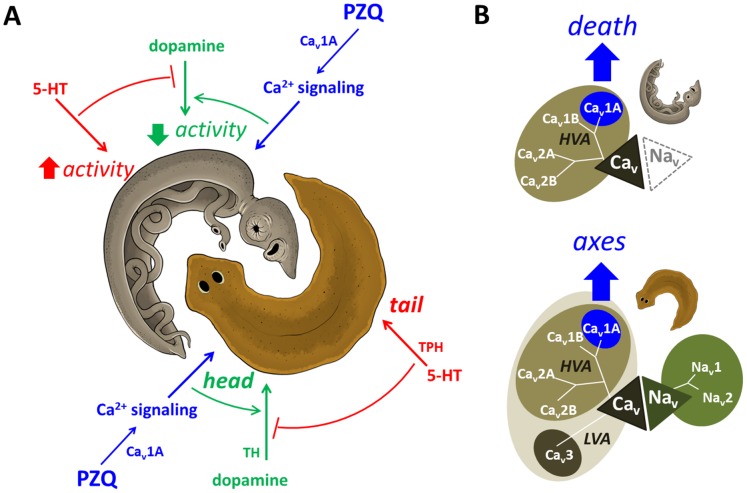
Death and axes: phenologous responses evoked by PZQ in different organisms. (**A**) Proposed model depicting phenology between PZQ-evoked outcomes in planarians (brown) and schistosomes (grey, adult worm depicted). In both organisms, we suggest PZQ evoked Ca^2+^ entry (blue) couples to dopaminergic signals that promote outcomes (green, head regeneration/paralysis) that are antagonized by serotonergic signals coupling to opposing phenotypes (red, tail regeneration/hyperactivity). (**B**) In planarians (bottom), a broad array of voltage-gated entry channels permits subfunctionalization of PZQ-evoked Ca_v_1A activity (blue) to yield a physiological exploitable Ca^2+^ influx. In contrast, schistosomes (top) are more vulnerable to PZQ-evoked Ca_v_1A activity, as these parasites possess a more limited repertoire of voltage-sensitive influx channels, lacking Na_v_ and LVA Ca_v_ channels. Sequence identifiers - *Dugesia japonica*: Ca_v_ 1A (AEJ87267), Ca_v_ 1A (AEJ87268), Ca_v_ 2A (AEJ87269), Ca_v_ 2B (AEJ87270), Ca_v_3 (AEJ87271), Na_v_1 (FY933419), Na_v_2 (FY957659). *Schistosoma mansoni*: Ca_v_ 1A (Smp_020270), Ca_v_ 1B (Smp_159990), Ca_v_ 2A (Smp_020170) & Ca_v_2B (Smp_004730).

Beyond the conservation of single genes as nodes in a signaling pathway, broader network architectures are conserved between diverse organisms. While the phenotypic outputs of these networks are diverse, their common architecture provides the mechanistic basis for predictive phenology [23]. We suggest these divergent PZQ-evoked outcomes (death versus axes) represent unexpected Ca^2+^-dependent phenologs initiated by small molecule activation of a signaling node (Ca_v_1A) within a shared bioaminergic interactome (Figure 5A). This conservation infers reciprocal predictive value for both discovery of new antischistosomal compounds, and reciprocally new signalling pathways impacting anterior-posterior signaling in planarians. We illustrate this principle here by highlighting *de novo* new compounds effective against schistosomules (bromocriptine) and new druggable targets (bioaminergic signaling) as the downstream PZQ-evoked interactome is revealed in the more tractable planarian model. PZQ engages similar pathways in these different platyhelminths such that chemical/functional genetic approaches in planarians can assist in discovering next generation antischistosomals and resolving their molecular action. This line of reasoning is analogous to a longer history of studies exploiting *C. elegans* for comparative insight into new drugs targeting parasitic nematodes, and this experience underscores both the utility of this approach but also the frustration in harvesting viable clinical leads from a large number of efficacious compounds in both nematode models [50], [51].

Reciprocally, this unexpected phenology can reveal new modulators of AP patterning from the schistosome screening literature (e.g. PKC, GSK3). Such insight from schistosome life cycle drug screens will be of utility for understanding the process of *in vivo* stem cell differentiation and CNS regeneration in response to injury that are inherent to the remarkable regenerative prowess of planarians. Indeed, resolution of the coupling of specific neuronal Ca_v_ channels to defined neurotransmitters integrates our studies of PZQ-evoked Ca_v_ activity [14], [15] with an older literature supporting a role for bioamines in planarian regeneration [52].

But how is small molecule activation of Ca_v_1A in one organism deleterious, but the same Ca^2+^ influx process harnessed physiologically in another to regulate polarity during regeneration? We speculate the same PZQ-evoked interactome differentially couples to these outcomes because of the different ionotropic channel portfolio supporting cellular excitability in the two organisms. Planarians express a surprisingly broad array of voltage-gated entry channels - five unique Ca_v_ channels in addition to Na_v_ channels (Figure 5B). This broad channel repertoire likely permits subfunctionalization of Ca_v_1A activity within a broad organismal complement of voltage-gated channels in planarians to yield a physiological exploitable Ca_v_1A dependent Ca^2+^ influx. In contrast, schistosomes express a more limited portfolio of voltage-sensitive channels, lacking both Na_v_ and LVA Ca_v_ channels (Figure 5B). The more limited gene repertoire of these parasites imparts a dependency and thereby vulnerability to Ca_v_1A activity within their smaller ionotropic channel portfolio. In this context, it is intriguing that both muscle contraction and tegumental damage are Ca^2+^ triggered phenomena in adult schistosomes (reviewed in [17]), such that Ca^2+^ dysregulation may serve as a common nexus predictive of *in vivo* antihelmintic activity. Further insight into this problem will be provided by understanding how acute Ca^2+^-dependent effects evoked by PZQ in different schistosome tissues regulate both acute downstream targets (bioaminergic receptors and their second messenger coupling) and the relevance of more chronic Ca^2+^ dependent transcriptional effects [20], [22], e.g. CamKII [21], that have emerged from recent mRNA profiling analyses.

In conclusion, exploitation of this Ca^2+^ dependent phenology should rekindle interest in drugs such as bromocriptine, and the druggability of their cognate bioaminergic receptors, as an avenue for resolving novel antischistosomals and modulating in vivo stem cell behavior during regeneration.

## Materials and Methods

### Planarian husbandry

A clonal line of *Dugesia japonica* (GI strain) was maintained at room temperature and fed strained chicken liver puree once a week [53]. Regenerative assays were performed using 5 day-starved worms in pH-buffered Montjuïch salts (1.6 mM NaCl, 1.0 mM CaCl_2_, 1.0 mM MgSO_4_, 0.1 mM MgCl_2_, 0.1 mM KCl,1.2 mM NaHCO_3_, pH 7.4 buffered with 1.5 mM HEPES) as described previously [53]. Drugs were sourced as indicated in Table 1, and used either at the highest concentrations which did not impact worm viability, or at lower concentrations if such treatments elicited an effect of maximal penetrance. Planarian regenerative phenotypes were archived using a Zeiss Discovery v20 stereomicroscope and a QiCAM 12-bit cooled color CCD camera.

### Cloning strategies and RNAi

Total RNA was isolated from 50 intact planarians using TRIzol® and poly-A purified using a NucleoTrap mRNA mini kit. cDNA was synthesized using the SuperScript™ III First-Strand Synthesis System (Invitrogen). Gene products were amplified by PCR (LA Taq™ polymerase), ligated into the pGEM®-T Easy vector (Promega) for sequencing, and subcloned into the IPTG-inducible pDONRdT7 RNAi vector transfected into RNase III deficient HT115 *E. coli*. *In vivo* RNAi was performed by feeding [53], and a *Schmidtea mediterranea* six-1 (*Smed*-six-1) construct, which did not yield a phenotype in *D. japonica*, was used as a negative control. RNAi efficiencies varied between different genes, but mRNA knockdown typically ranged anywhere between 20–80%. Targeted sequences: tyrosine hydroxylase (NCBI accession numbers AB266095.1, 136–1657 bp), tryptophan hydroxylase (AB288367.1, 4–1623 bp), tyramine beta-hydroxylase (671–1629 bp), tyrosine/histidine decarboxylase (FY934632.1, 26–685 bp), glutamate decarboxylase (AB332029.1, 154–1937 bp), choline acetyltransferase (AB536929.1, 74–1175 bp), prohormone convertase 2 (PC2 (1–2285 bp), Ca_v_1A (HQ724315.1, 2229–4133 bp), *Smed*-six-1 (AJ557022.1, 1–506 bp). Protein kinase C (PKC) sequences and DAGK were cloned from planarian ESTs displaying homology to *Schistosoma mansoni* PKC isoforms - cPKC (FY950278.1, FY947802.1, FY970060.1), aPKC (FY933556.1, FY941429.1), nPKC (FY934640.1) and DAGK (FY953983, FY959647.1, and BP187372.1).

### Schistosomule isolation

*Biomphalaria glabrata* snails exposed to mirarcidia (NMRI Puerto Rican strain of *Schistosoma mansoni*) were obtained from the Biomedical Research Institute (Rockville, MD) and maintained at 26°C for 4 to 6 weeks. Matured cercaria were shed into aged tap water (40 ml) by exposure to light (1.5 hrs) and subsequently transformed into schistosomules [54]. Briefly, cercaria were separated from debris by filtration (47 µm) and then captured onto a 25 µm filter prior to resuspension in aged tap water with an equal volume of DMEM. Cercaria tails were sheared by three rounds of vortexing (45 sec), each followed by incubation on ice (3 min) prior to tail removal by Percoll column centrifugation (24 ml Percoll, 4 ml 10× Eagle's minimum essential medium, 1.5 ml penicillin-streptomycin, ml of 1M HEPES in 0.85% NaCl, 9.5 ml distilled water) at 500 g (15 mins, 4°C). The tail-containing supernatant was discarded and the pellet-containing bodies were washed three times in DMEM (400 g, 10 mins), resuspended in modified Batch's media [55] and transformed into schistosomules (incubation at 37°C/5% CO_2_).

### Schistosomule contractility assays

For contractility assays, drugs were solubilized in DMSO and diluted in pre-warmed modified Batch's media. While detailed protocols for quantifying aspects of worm dynamics in adult worms [21], or higher throughput screening of schistosomules [56] have been developed, the effects on schistosomule activity were simply quantified here using a custom written plugin (wrMTrck) in ImageJ to using resolve schistosomule body length (major axis of an ellipse) over time following drug exposure (30 min), just as in [39].Videos were captured using a Nikon Coolpix 5700 camera affixed to a Nikon Eclipse TS100 microscope. Typically, for a single video ∼7–10 schistosomula were measured within the field of view (10× microscope objective) over a 2 minute recording period. Data represent means for analysis of results from three independent treatments.

### Radioligand binding assays

Planarian membrane fractions were prepared by homogenizing worms on ice (∼1000 worms/prep) in HEPES (20 mM) supplemented with cOmplete™ protease inhibitor cocktail (Roche). Cellular debris was pelleted by centrifugation (8000 g for 5 mins) and the resulting supernatant was centrifuged (56,000 g for 45 mins) to yield a pelleted membrane fraction. This material was resuspended (20 mM HEPES, with protease inhibitors) to a final protein concentration of ∼5 µg/µl and stored at −80°C. Binding assays were performed on planarian membrane protein (50 µg) with 26 nM ^3^H-dopamine (specific activity 21.2 Ci/mmol, Perkin Elmer). Indirect binding assays were performed with various ligands (bromocriptine, 10 µM; haloperidol, 100 µM; apomorphine, 10 µM) in TE buffer (1 mM EDTA, 50 mM Tris-HCl, pH 8.3; final volume of 500 µl). Samples were incubated on ice for 15 minutes, after which time 500 µl PEG (30%) and 20 µL IgG (25 mg/ml) were added and samples centrifuged (20,000 g for 5 mins). The resulting pellet was washed (PEG, 15%), centrifuged (20,000 g for 5 mins) and solubilized in TE (200 µL, containing 2% Triton X-100). Displacement was measured by liquid scintillation counting and nonspecific binding assessed by subtraction of values in samples incubated with cold dopamine (1 mM). All centrifugation steps were performed at 4°C.

### HPLC analysis of neurotransmitters

Thirty planarian trunk fragments were amputated and incubated with or without specific drugs for 24 hrs, after which time media was removed and replaced with ascorbic acid (300 µl, 1% m/v). Samples were then lysed by three successive freeze-thaw cycles and cellular debris pelleted by centrifugation (10,000 g for 5 mins). The resulting supernatant was then filtered (0.45 µm filter plate, Millipore) by centrifugation (3,000 g for 10 mins) and the filtrate (180 µL) supplemented with 0.5M HClO_4_ (20 µl, 500 mM final concentration). The samples were mixed and injected by an autosampler into an Agilent 1200 HPLC apparatus, with a 5 µm, 4.6×150 mm Eclipse XDB C18 column attached to a Waters 2465 electrochemical detector with a glassy carbon-based electrode. The current range was set at 50 nA with a working potential of 0.7 V versus an *in situ* Ag/AgCl reference electrode. The mobile phase mixture (13 mg/L of the surfactant sodium octylsulfate, 170 µL/L dibutylamine, 55.8 mg/L Na_2_EDTA, 10% methanol, 203 mg/L sodium acetate anhydrous, 0.1M citric acid, and 120 mg/L sodium chloride) was ran at a flow rate of 2 ml/min. The area underneath the peaks was analyzed for total amount of serotonin and dopamine. Results were normalized to sample protein concentration determined by Bradford assay (Thermo Scientific).

### Data analysis

Data were analyzed using two-tailed, unpaired t-tests, and presented as mean ± standard error of the mean from at least three independent assays, except where indicated. Differences were considered significant at p<0.05 (*), p<0.01 (**).
